# Ultrasound-Guided Anterior Quadratus Lumborum Block Reduces Postoperative Opioid Consumption and Related Side Effects in Patients Undergoing Total Hip Replacement Arthroplasty: A Propensity Score-Matched Cohort Study

**DOI:** 10.3390/jcm10204632

**Published:** 2021-10-09

**Authors:** Yeon-Ju Kim, Hyung-Tae Kim, Ha-Jung Kim, Pil-Whan Yoon, Ji-In Park, Sun-Hyung Lee, Young-Jin Ro, Won-Uk Koh

**Affiliations:** 1 Department of Anesthesiology and Pain Medicine, Asan Medical Center, University of Ulsan College of Medicine, 88 Olympic-ro 43-gil, Songpa-gu, Seoul 05505, Korea; yjk88@amc.seoul.kr (Y.-J.K.); ingwei2475@gmail.com (H.-T.K.); alexakim06@gmail.com (H.-J.K.); monica0120120@gmail.com (J.-I.P.); yjro@amc.seoul.kr (Y.-J.R.); 2 Department of Orthopedic Surgery, Asan Medical Center, University of Ulsan College of Medicine, 88 Olympic-ro 43-gil, Songpa-gu, Seoul 05505, Korea; orthoyoon@amc.seoul.kr (P.-W.Y.); Sunhyung123@gmail.com (S.-H.L.)

**Keywords:** pain, quadratus lumborum block, total hip arthroplasty, ultrasound-guided

## Abstract

Quadratus lumborum block (QLB) has been shown to be effective for pain relief after hip surgery. This study evaluated the efficacy of ultrasound-guided anterior QLB in pain control after total replacement hip arthroplasty (TRHA). A total of 115 patients receiving anterior QLB were propensity score-matched with 115 patients who did not receive the block. The primary outcome was opioid consumption at 24, 24–48, and 48 postoperative hours. Secondary outcomes included pain scores at the post-anesthesia care unit (PACU), 8, 16, 24, 32, 40, and 48 h length of hospital stay, time to first ambulation, and the incidence of opioid-related side effects. Postoperative opioid consumption 48 h after surgery was significantly lower in the QLB group. Resting, mean, worst, and the difference of resting pain scores compared with preoperative values were significantly lower in the QLB group during the 48 postoperative hours. The length of hospital stay was shorter in the QLB group. The incidence of postoperative nausea and vomiting was significantly lower in the QLB group during the 48 postoperative hours, except at the PACU. This study suggests that anterior QLB provides effective postoperative analgesia for patients undergoing THRA performed using the posterolateral approach.

## 1. Introduction

Total hip replacement arthroplasty (THRA) has been shown to improve long-term quality of life, although it can cause moderate to severe perioperative pain [1]. Adequate postoperative analgesia is important because it has been associated with increased patient satisfaction, earlier mobilization, and decreased length of hospital stay. Traditionally, perioperative pain has been managed by epidural analgesia, parenteral opioids, and peripheral nerve blocks. Although epidural analgesia is efficacious [2], it is generally difficult to apply because of its side effects and rare but major complications [3]. Peripheral nerve blocks are known to cause fewer serious side effects than epidural anesthesia [4]; therefore, several techniques such as femoral nerve block or lumbar plexus block have been introduced as possible alternatives of epidural analgesia for pain control after THRA [5]. However, optimal regional analgesic intervention for THRA is yet to be defined because innervation of the hip joint is complex and preservation of lower extremity motor function is further needed for early mobilization and prompt recovery in current practice guidelines [6]. 

Quadratus lumborum block (QLB) is a relatively new truncal regional block technique that has the potential to alleviate somatic as well as visceral pain after abdominal surgery [7]. This fascial plane block aims to anesthetize the thoracolumbar nerves by injecting local anesthetics around the quadratus lumborum muscle [8]. There are several approaches based on injection location to the QL block: lateral, posterior, and anterior QLB. There is a difference in mechanism depending on the type of approach; accordingly, a different QLB is applied for each operation. Case studies have recently reported that QLB has an analgesic effect on the hip joint [9], and its effectiveness has been demonstrated [10]. The pathway of the anterior (or transmuscular) QLB injectate can potentially spread to the paravertebral (PVB) space with coverage of the nerves providing sensory innervation to the hip [11]. Furthermore, this block has the added benefit of minimizing quadriceps weakness [12]. 

The purpose of this study was to evaluate the efficacy of ultrasound-guided anterior QLB in pain control after THRA by comparing the outcome of opioid consumption between two groups: patients undergoing THRA with QLB (QLB group) and patients undergoing THRA without QLB (control group). In addition, we measured the rate of postoperative opioid-related side effects, including nausea, vomiting, hypotension, and urinary retention, as secondary outcomes. 

## 2. Materials and Methods

### 2.1. Participants

The protocol of this retrospective cohort study was approved by the institutional review board of Asan Medical Center (2021-0461). This study included a single surgeon’s (PWY) consecutive series of patients who were scheduled for primary unilateral THRA between January 2019 and February 2021. We started performing QLB in our institute from February 2020 onward in THRA patients who gave their consent to receive the block. A total of 128 patients received QLB between February 2020 and February 2021, and 61 declined to receive the block. The 128 patients who received the block were designated to the QLB group, and the 61 patients who did not receive the block, along with 112 patients between January 2019 and January 2020, were assigned to the control group. The requirement of informed consent was waived in this study because the data were collected by reviewing electronic medical records. Patients were included in the study if they met the following eligibility criteria: age > 18 years, American Society of Anesthesiologists (ASA) physical status classification I–III, and scheduled for elective primary unilateral THRA. The exclusion criteria included patients with chronic pain or daily opioid consumption before surgery exceeding that of chronic opioid users, patients who were transferred to the intensive care unit (ICU) after surgery or remained sedated, emergency surgeries, revision surgeries, patients who did not receive intravenous patient-controlled analgesia (IV PCA), and patients with incomplete medical records. 

### 2.2. Quadratus Lumborum Block 

**Figure 1:**
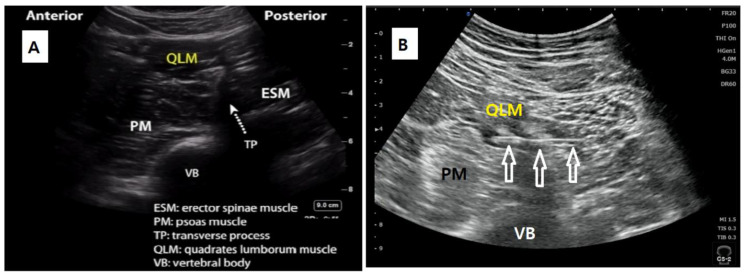
Schematic illustration of the procedure of QLB. (**A**) The Shamrock sign; (**B**) ultrasound image of anterior QLB with displaying needle trajectory (white arrows display needle trajectory). QLM, quadratus lumborum muscle; PM, psoas muscle; TP, transverse process; ESM, erector spinae muscle; VB, vertebral body.

Anterior QLB was performed preoperatively in a separate block room using the ultrasound-guided posterior approach as previously described [13,14]. Patients were placed in the lateral decubitus position with the surgical side upward. A low frequency convex ultrasound transducer (5-2 MHz probe, Sonimage HS1, Konica Minolta Inc. Tokyo, Japan) was placed in the mid-to-posterior axillary line and between the costal margin and iliac crest. After obtaining the “Shamrock” view (Figure 1) in the L3 vertebral level, a 21 gauge 100–120 mm block needle (Echoplex^®^, Vygon, Ecouen, France) was inserted in-plane from the posterior edge of the convex probe and advanced through the quadratus lumborum muscle in a posterior-to-anterior direction until the needle tip was placed between the fascial interspace of the psoas major muscle and quadratus lumborum muscle. A total of 25–35 mL of 0.3% ropivacaine was injected in the fascial interspace with intermittent aspiration to confirm the absence of blood. Successful injectate spread was confirmed by visualization of the separation of the quadratus lumborum and psoas muscles in the axial plane, with further identification of the caudal and cephalad injectate spread from the iliac crest toward the diaphragm.

### 2.3. Anesthesia and Perioperative Management

Every patient received 400 mg of oral celecoxib immediately before entry to the operation theatre, and no other premedication was given. After the patient arrived in the operating theatre, standard monitoring, including noninvasive blood pressure monitoring, electrocardiography, and pulse oximetry, was applied. Most of the patients received spinal anesthesia as the main anesthetic, although general anesthesia was also applied in some cases at the discretion of the attending anesthesiologist. Spinal anesthesia was performed in the lateral decubitus position with the surgical limb facing downward, using hyperbaric 0.5% bupivacaine (standard dose 10–15 mg), with the addition of 10–15 mcg of fentanyl. Once the spinal anesthetic level was confirmed, patients received intravenous sedation with a target-controlled infusion (TCI) of propofol (0.5–2 μg/mL). All patients breathed spontaneously with supplemented oxygen of 6 L/min via a simple facial mask. In case of general anesthesia, induction was performed with a bolus injection of propofol 1.5–2 mg/kg, remifentanil TCI 2–3 ng/mL, and rocuronium 0.6–0.8 mg/kg; tracheal intubation was also performed. General anesthesia was maintained using desflurane 5–6%, air, oxygen, and remifentanil TCI 1.5–4.0 ng/m. Every patient received 0.3 mg of intravenous ramosetron for antiemetic prophylaxis following anesthetic induction. 

All THRAs were performed using a posterolateral approach in the lateral position with the operative side facing upward. After completion of the arthroplasty and joint capsule closure, local anesthetic injection (0.5% bupivacaine 100 mg) to the joint cavity was performed by the surgeon to every THRA patient as part of a multimodal analgesic strategy. There were no significant changes to surgical practice or local injection strategy of the surgeon during the study period. After surgery, patients were transferred to the post-anesthesia care unit (PACU) for post-anesthetic recovery. They were later transferred to the general ward after achieving an Aldrete’s score of 9–10. 

### 2.4. Postoperative Analgesia

All study patients regardless of study group received a standardized regimen of 200 mg of oral celecoxib daily and an IV PCA comprising fentanyl doses of approximately 0.2 mcg/kg/dose for up to a maximum of six doses per hour for postoperative pain management. Basal infusions were not programmed in the PCA infuser pump. The amount of PCA used was recorded by the pain management service and ward nursing staff every 8 h after surgery. Further rescue doses of either tramadol (1 mg/kg/dose) or hydromorphone (0.02 mg/kg/dose) were administered on demand depending on the extent of breakthrough pain at the discretion of the attending physician. Postoperative resting, mean, and worst pain scores were recorded after surgery by the nursing staff using a 10-point numerical rating scale (NRS: 0, no pain; 10, worst imaginable pain ever) at the PACU and general ward every 8 h. If there was breakthrough pain that required additional rescue analgesia, the event and pain score were also recorded. In case of postoperative nausea or vomiting, the event was recorded, and a rescue dose of 0.3 mg ramosetron was administered intravenously by the nursing staff. This standardized pain management protocol was not changed during the entire study period. 

### 2.5. Statistical Analyses

We performed propensity score-matching on the two groups to reduce any potential impact of treatment selection bias or covariates on the study outcome. Patients in the QLB and control groups were matched using propensity scoring in a 1:1 ratio. The propensity score was estimated with QLB as the dependent variable by multiple logistic regression analysis. A full non-parsimonious model was developed that included age, gender, body mass index (BMI), ASA class, diabetes, hypertension, coronary artery disease, cerebrovascular disease, anesthetic method, preoperative pain score, and the interaction terms between variables. Absolute standardized differences were used to diagnose the balance. All absolute standardized differences after matching were <0.1. 

Variables with non-skewed distributions were reported as means (standard deviations), and differences were evaluated using the unpaired Student’s t-test. Continuous variables with skewed distributions were reported as medians (interquartile ranges), and differences were assessed using the Mann–Whitney test. Categorical variables were reported as numbers and percentages, and differences were evaluated using the chi-square test or Fisher’s exact test.

After matching, the primary outcome was opioid consumption (calculated as IV morphine equivalents) at 24, 24–48, and 48 postoperative hours, which was compared using a Wilcoxon signed-rank test or paired t-test. Intraoperative opioids were converted to IV morphine equivalents for analysis using previously established conversion ratios [15]. Secondary outcomes included NRS pain scores (resting, mean, and worst) at the PACU, 8, 16, 24, 32, 40, and 48 h after surgery, length of hospital stay (from the time of operation to the time of hospital discharge), and time to first ambulation (the time from arrival in the PACU until first ambulation). The incidence of opioid-related side effects, including postoperative nausea and vomiting (PONV), amount of rescue antiemetics, incidence of urinary retention, respiratory depression, and hypotension, was compared between the two groups using a conditional logistic regression model. Other side effects, including delirium, surgery-related complications, fever, and renal or hepatic dysfunction, were also compared. 

## 3. Results

**Figure 2:**
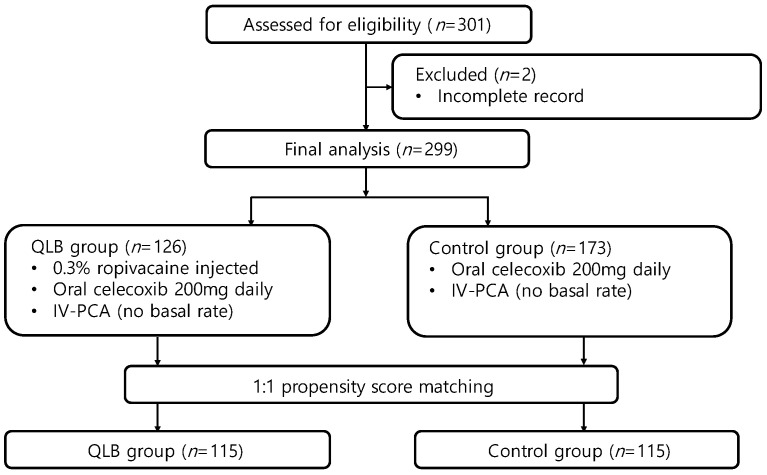
Flow chart of patient selection and propensity score-matching. QLB, quadratus lumborum block; PCA, patient-controlled analgesia; IV, intravenous.

The initial search strategy identified a cohort of 301 patients (128 in the QLB group and 173 in the control group) for chart review. Two patients had incomplete medical records with missing key data points (two in the QLB group). Following propensity score-matching, 115 patients who received QLB and 115 patients who did not receive a block were ultimately analyzed (Figure 2).

Patient sex, age, BMI, ASA score, anesthetic and operative time, and preoperative NRS score were similar between both groups (Table 1). After propensity score-matching, 109 patients received spinal anesthesia and 6 patients received general anesthesia in the QLB group, and 111 and 4 patients received spinal and general anesthesia in the control group, respectively.

**Figure 3:**
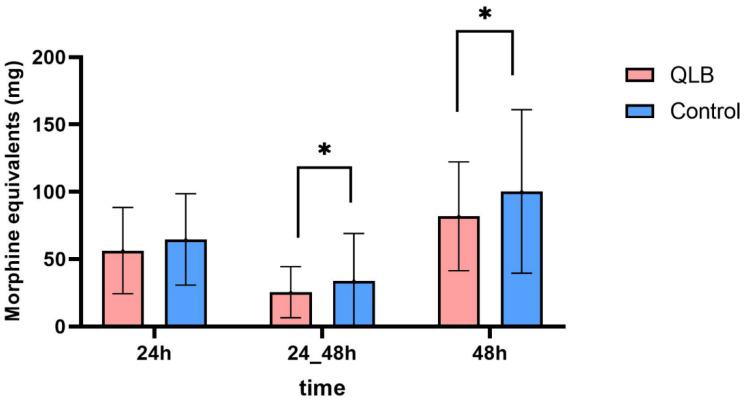
Postoperative opioid consumption during the 48 h postoperative period between the QLB and control group. Values are mean ± SD. * *p* < 0.05 QLB, quadratus lumborum block.

There was no significant difference in postoperative rescue opioid consumption in the 24 h postoperative period between the groups (56.3 mg (34.5–75) vs. 64.6 mg (40.1–78.), *p* = 0.06). However, opioid consumption was significantly lower in the QLB group in the 24–48 h postoperative period (23.45 mg (9–39) vs. 35.6 (12–47.8), *p* = 0.01). In addition, total opioid consumption 48 h after surgery was significantly lower in the QLB group than in the control group (81.8 mg (52–112.5) vs. 100.3 mg (64.2–124.8), *p* = 0.02) (Figure 3). The time to the first rescue opioid intake was not significantly different between the groups (765.6 min (330–1440) vs. 525.6 min (210–1260), *p* = 0.13).

**Figure 4:**
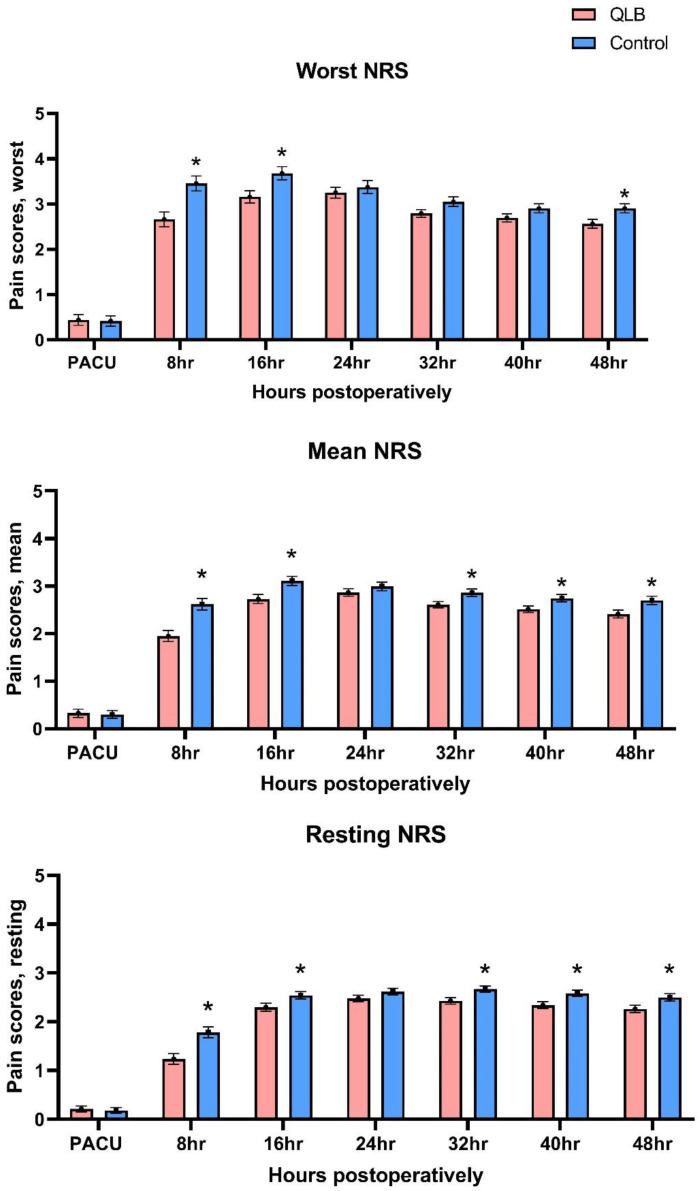
Comparison of pain intensity in the PACU at 8, 16, 24, 32, 40, and 48 h after surgery between QLB and Control groups. Values are mean ± SD. * *p* < 0.05 PACU, post-anesthesia care unit; NRS, numeral rating scale; QLB, quadratus lumborum block.

Resting, mean, and worst pain scores compared with preoperative values were significantly lower in the QLB group during the 48 postoperative period (Figure 4) (*p* = 0.041, 0.007, and 0.020 respectively). Pain scores and difference of resting pain compared to preoperative pain scores for each group at different time points are shown in the supplementary materials (Table S1). 

The length of hospital stay was shorter in the QLB group than in the control group (5.1 ± 1.4 days vs. 5.8 ± 2.9 days, *p* = 0.025). However, there was no significant difference in the time to first ambulation between the two groups (49.0 ± 15.3 h vs. 48.9 ± 14.7 h, *p* = 0.961). The incidence of PONV and amount of antiemetics were significantly lower in the QLB group during the 48h postoperative period (Table 2). There were no significant statistical differences in the incidence of urinary retention, respiratory depression, and hypotension between the two groups (Table 2). 

No statistical intergroup differences were observed for any of the other side effects of postoperative delirium, surgery-related complications, postoperative fever, and renal or hepatic dysfunction. 

## 4. Discussion

In this retrospective propensity score-matched cohort study, ultrasound-guided anterior QLB provided effective postoperative analgesia after elective THRA, resulting in a significant reduction of opioid consumption within the 48h postoperative period. Pain scores in our treatment cohort were significantly lower during the 48h postoperative period, with a significant difference at each other measured time point. Additionally, we found that anterior QLB significantly reduced the incidence of PONV, the use of antiemetics, and could lead to a faster recovery in the early postoperative phase. 

It is well known that QLB evolved from the transversus abdominis plane block, which provides adequate analgesia to a patient when undergoing abdominal surgery [16,17]. However, there is increasing evidence that QLB can also act on the branches of the lumbar plexus and provide analgesia in hip surgery. QLBs are classified into lateral, posterior, and anterior, based on the anatomical location of needle tip placement in relation to the quadratus lumborum muscle [8]. Previous cadaveric studies reported that different QLBs have different mechanisms of action [18]. Posterior QLBs are associated with injectate spread along the middle thoracolumbar fascia intertransverse area [11], and lateral QLB injectate may spread to the transversus abdominis muscle plane [19]. Thus, lateral and posterior QLBs may generate analgesia from T8 to L1 and play a role in postoperative pain management for abdominal surgery [16]. However, previous studies have shown that posterior QLB is ineffective for postoperative pain control in hip surgery [20]. Regarding the anterior QLB examined in this study, the local anesthetic may spread to the lumbar plexus nerves and branches in addition to the thoracic paravertebral space [7]. Therefore, anterior QLB may provide analgesia not only to the trunk but also to the lower extremities from T10 to L4 [19,21]. For this reason, anterior QLB has been considered as a motor-sparing alternative to lumbar plexus block [22]. The nociceptors of the hip capsule are mainly situated in the anterolateral part of the capsule, which is innervated by the femoral and obturator nerves [23]. Thus, the spread of local anesthetic in the fascial plane between the quadratus lumborum and psoas major muscles in anterior QLB enables the block of the branches of the lumbar plexus, which include the femoral, obturator, and lateral femoral cutaneous nerves. In this study, the local anesthetic infiltration to the joint cavity via the posterior joint capsule performed by the surgeon may have further contributed to this result by blocking the branches arising from the sacral plexus.

In a randomized study, Kukreja et al. compared single-shot anterior QLB with no block in patients undergoing THRA. Anterior QLB provided effective analgesia and decreased opioid requirements compared with patients receiving no block [24]. In another report, transmuscular QLB employment in hip surgery patients significantly reduced the length of hospital stay and use of intraoperative fentanyl [25]. This study also demonstrated consistent results with previous studies by showing significant reduction in postoperative opioid consumption and pain scores, suggesting that anterior QLB can be an effective pain management modality in THRA patients.

Another noteworthy outcome in this study is that the incidence of PONV and use of antiemetics were significantly reduced in the QLB group compared with the control group. Although the occurrence of PONV is a complex, multifactorial problem [26], the decrease in PONV in the QLB group appears to be the result of the reduction in postoperative opioid use compared with the control group. An increase in opioid consumption is associated with side effects such as nausea, vomiting, and gastrointestinal paralysis, which can be detrimental to patients’ recovery after surgery [27]. These side effects occur when opioids act on presynaptic receptors in the myenteric nerve plexus, leading to an increase in the non-propulsive contraction of the bowel [28]. In addition, continuous higher pain scores in the control group may have further contributed to the difference. This decrease in PONV incidence along with lower pain scores in the QLB group appears to have contributed to the decreased length of hospital stay of the QLB group compared with the control group.

There is no current consensus on optimal regional analgesic techniques after THRA [29]. Various regional techniques for THRA have been introduced for postoperative analgesia, which include lumbar plexus block, femoral nerve block, sciatic nerve block, fascia iliaca compartment block, and pericapsular nerve group (PENG) block. The selection of each block depends on patient status, surgical approach, and preference of the attending anesthesiologist, and each technique has its own respective advantages and disadvantages. For example, the analgesic efficacy of lumbar plexus block in decreasing pain scores and opioid consumption has been widely described [29]. However, the incidence of major complications, including epidural or intrathecal spread of local anesthetics and visceral injury, has been reported along with quadriceps weakness, which may delay the time of ambulation after surgery [30]. The efficacy of femoral nerve block for analgesia after hip surgery is unclear; thus, its routine use after TRHA is not recommended [31]. Previous studies have reported that fascia iliaca block did not improve analgesia after hip arthroscopy surgery, but rather resulted in quadriceps weakness, which may contribute to an increase in fall risk and delayed rehabilitation [32]. The PENG block is a novel technique for the blockade of the articular branches that innervate the anterior hip capsule [33]. Previous studies have reported that the PENG block not only provides effective analgesia but also preserves motor fibers to enable early ambulation in hip surgery patients [34,35]. However, as the number of related studies remains small, further evaluation is needed. Due to these concerns, alternative peripheral nerve block targets have been investigated. Several recent studies have shown that QLB could be successfully used for hip surgeries with several advantages [24,25,36]. Our study found that QLB with periarticular local infiltration in THRA conducted using the posterior approach reduces postoperative opioid requirements and the length of hospital stay without prolongation of ambulation time. However, the effect of each block may present different results depending on the type of surgical approach used for THRA, and large-scale studies comparing various regional techniques according to surgical manipulation are needed in the future to identify the optimal technique for THRA.

There are some limitations present in this study. First, this study had a retrospective and non-randomized design, which has a possibility of selection bias. In addition, several patients were excluded from analysis due to missing data. However, we compensated for this gap with the use of propensity score-matching, which creates groups that are similar with respect to covariates. Second, related to the retrospective nature of our study, outcomes such as quadriceps weakness, dynamic pain scores, and patient satisfaction were not recorded; therefore, these outcome measures could not be addressed. This lack of data certainly limits the strength of this retrospective study. Third, every surgery in this study was performed using the posterolateral approach; thus, the results of this study may not apply to THRAs that are performed using lateral or anterior approach techniques.

## 5. Conclusions

Anterior QLB provides effective postoperative analgesia for patients undergoing THRA performed using the posterolateral approach. Proper implementation of the technique can significantly decrease opioid consumption and pain scores, as well as length of hospital stay, without delaying time of ambulation. In addition, the incidence of PONV, an opioid-related side effect, was significantly reduced. Further studies are required to investigate optimal block methods according to surgical approach technique.

## Figures and Tables

**Table 1:** Patient characteristics and perioperative variables.

	Before Matching				After PS Matching		
	QLB (*n* = 126)	Control (*n* = 173)	*p*-Value	SMD	QLB (*n* = 115)	Control (*n* = 115)	SMD
Age (year)	54.3 ± 13.3	58.6 ± 17.0	0.015	−0.279	54.9 ± 13.5	54.1 ± 16.8	0.051
Gender, male	66 (52.3)	77 (44.5)	0.178	0.158	56 (48.7)	59 (51.3)	−0.052
BMI (kg/m^2^)	25.5 ± 3.6	24.5 ± 3.9	0.037	0.245	25.2 ± 3.6	24.9 ± 3.8	0.097
ASA PS I/II/III	7.94/86.51/5.56	8.67/75.14/16.19	0.022	0.303	8.7/85.22/6.09	7.83/86.96/5.22	0.058
HTN	39 (30.95)	76 (43.93)	0.022	−0.270	38 (33.04)	37 (32.17)	0.018
DM	16 (12.7)	28 (16.18)	0.400	−0.099	14 (12.17)	13 (11.3)	0.027
IHD	5 (3.97)	9 (5.2)	0.617	−0.059	3 (2.61)	2 (1.74)	0.059
CVA	6 (4.76)	12 (6.94)	0.435	−0.122	6 (5.22)	6 (5.22)	0
Preoperative NRS	2.59 ± 0.69	2.54 ± 0.82	0.897	0.055	2.61 ± 0.66	2.66 ± 0.67	−0.071
Duration of anesthesia (min)	147.34 ± 25.48	141.49 ± 28.22	0.066	0.217	145.86 ± 26.02	139.34 ± 21.79	0.271
Duration of surgery (min)	94.84 ± 26.29	95.31 ± 30.41	0.888	−0.016	93.65 ± 26.13	95.10 ± 25.13	−0.056
Anesthetic method Spinal anesthesia General anesthesia							
	120 (95.24)	152 (87.86)	0.028	0.267	109 (94.78)	111 (96.52)	−0.071
	6 (4.76)	21 (12.14)			6 (5.22)	4 (3.48)	

Results are expressed as mean ± SD, median [IQR], *n* (%). QLB, quadratus lumborum block; BMI, body mass index; ASA PS, American Society of Anesthesiologists physical status; HTN, hypertension; DM, diabetes mellitus; IHD, ischemic heart disease; CVA, cerebrovascular accident; NRS, numeral rating scale; SMD, standardized mean difference.

**Table 2:** Incidence of adverse events.

	QLB (*n* = 115)	Control (*n* = 115)	*p*-Value
Opioid related adverse events			
Nausea and vomiting			
24 h	13 (11.3)	50 (43.48)	<0.0001
48 h	15 (13.04)	52 (45.22)	<0.0001
Use of antiemetics			
24 h	22 (19.13)	73 (63.48)	<0.0001
48 h	23 (20)	73 (64.35)	<0.0001
Hypotension			
24 h	2 (1.74)	7 (6.09)	0.1104
48 h	3 (2.61)	9 (7.83)	0.0899
Urinary retention	46 (40)	38 (33.04)	0.2738
Other adverse events			
Hepatic dysfunction	5 (4.35)	6 (5.22)	
Pulmonary complication	0 (5)	11 (15.9)	
Op-related complication	2 (1.6)	1 (1.4)	
Fever	0 (0)	2 (1.74)	
Pruritis	0 (0)	2 (1.74)	

Results are expressed as *n* (%). QLB, quadratus lumborum block.

## Supplementary Materials

The following are available online at https://www.mdpi.com/article/10.3390/jcm10204632/s1, Table S1: The pain scores for each group at different timepoints.
