# Supplementary material for: Ultrasound-Guided Anterior Quadratus Lumborum Block Reduces Postoperative Opioid Consumption and Related Side Effects in Patients Undergoing Total Hip Replacement Arthroplasty: A Propensity Score-Matched Cohort Study
Source: J Clin Med. 2021 Oct 9;10(20):4632. doi: 10.3390/jcm10204632 (PMC8539613; doi:10.3390/jcm10204632)
Supplement: Supplementary file 1 [file jcm-10-04632-s001.zip › jcm-1364158-supplementary.pdf]

Table S1. The pain scores for each group at different timepoints.

		PACU		8 hr		16 hr		24 hr		32 hr		40 hr		48 hr		p for interaction
	Group	Lsmeans	SE	Lsmeans	SE	Lsmeans	SE	Lsmeans	SE	Lsmeans	SE	Lsmeans	SE	Lsmeans	SE	
Worst	QLB	0.4435	0.1186	2.6609	0.1624	3.1565	0.1362	3.2522	0.1197	2.7913	0.08601	2.6957	0.08922	2.5652	0.09968	***0.041***
	Control	0.4174	0.1152	3.4522	0.164	3.6783	0.1464	3.3739	0.1411	3.0522	0.1083	2.9043	0.09956	2.9043	0.1271	
	group p-value	0.8748		***0.0007***		***0.0097***		0.5111		0.0605		0.1199		***0.0369***		
Mean	QLB	0.3261	0.08691	1.9478	0.1163	2.7261	0.09512	2.8652	0.07941	2.6087	0.06616	2.513	0.06806	2.413	0.08129	***0.0075***
	Control	0.3	0.08327	2.6174	0.1201	3.1087	0.09463	2.9957	0.09129	2.8609	0.07577	2.7435	0.07722	2.7	0.09009	
	group p-value	0.8286		***<.0001***		***0.0047***		0.2821		***0.0129***		***0.0261***		***0.0189***		
Resting	QLB	0.2087	0.05987	1.2348	0.1121	2.2957	0.08191	2.4783	0.06576	2.4261	0.06658	2.3391	0.06638	2.2609	0.0742	***0.0204***
	Control	0.1826	0.05869	1.7826	0.1137	2.5391	0.07542	2.6174	0.06718	2.6696	0.06264	2.5826	0.06763	2.4957	0.07551	
	group p-value	0.7547		***0.0005***		***0.0417***		0.1582		***0.0134***		***0.0181***		***0.0431***		
Difference of resting (-Pre value)	QLB	-2.4043	0.09664	-1.3783	0.1364	-0.3174	0.09983	-0.1348	0.08475	-0.187	0.0792	-0.2739	0.0789	-0.3522	0.08099	***0.0145***
	Control	-2.4783	0.08764	-0.8783	0.126	-0.1217	0.09499	-0.04348	0.08229	0.008696	0.07956	-0.07826	0.08659	-0.1652	0.08815	
	group p-value	0.5716		***0.0076***		0.157		0.4404		0.0827		0.0963		0.1197		

Results are expressed as mean ± SE. LSmeans, least square means; QLB, quadratus lumborum block; PACU, post-anesthesia care unit.
